# Left bundle branch pacing in a 4-year-old patient with complete heart block after correction of transposition of the great arteries via a dual chamber pacing system: a case report

**DOI:** 10.3389/fcvm.2025.1587957

**Published:** 2025-06-10

**Authors:** Omar Fakhreddine, Patrick Sarkis, Jamil Francis, Bernard Abi-Saleh

**Affiliations:** Division of Cardiology, Department of Internal Medicine, American University of Beirut Medical Center, Beirut, Lebanon

**Keywords:** pacemaker, left bundle branch pacing, conduction system, conduction system abnormalities, congenital heart anomalies

## Abstract

Conduction system pacing is a recently developed pacing modality that serves as an alternative approach in maintaining or restoring physiological ventricular activity. Left bundle branch pacing (LBBP) has been rarely performed in children due to concerns regarding its safety and feasibility. Here, we report a case of successful LBBP in a 4-year-old patient with complete heart block following repair of transposition of the great arteries.

## 1 Introduction

Cardiac pacing is a well-known effective solution for symptomatic bradycardia caused by sinus node dysfunction or atrioventricular block. However, the traditional right ventricular apical pacing has been linked to atrial arrhythmias, left ventricular systolic dysfunction, and even increased mortality, which result from the electric and mechanical dyssynchrony it causes (1).

Conduction system pacing has been developed as an alternative approach in maintaining or restoring physiological ventricular activation, mainly by pacing the His bundle or the left bundle branch area (2).

Left bundle branch pacing (LBBP) has been rarely performed in children, with data on its safety and feasibility being limited to a few case reports (3).

Here, we report a case of successful left bundle branch pacing in a 4-year-old patient with complete heart block following repair of transposition of the great arteries (TGA).

## 2 Case presentation

We present the case of a 4-year-old boy born with transposition of TGA with interventricular communication (IVC) and right ventricular outflow tract obstruction. At 2 months of age, he underwent a Blalock–Taussig (BT) shunt insertion followed by a Nikaidoh procedure with takedown of the Blalock–Taussig shunt at the age of 1 year.

Unfortunately, his postoperative course was complicated by complete heart block requiring the implantation of a left epigastric dual chamber pacemaker with epicardial leads 3 days following the surgery.

**Figure 1:**
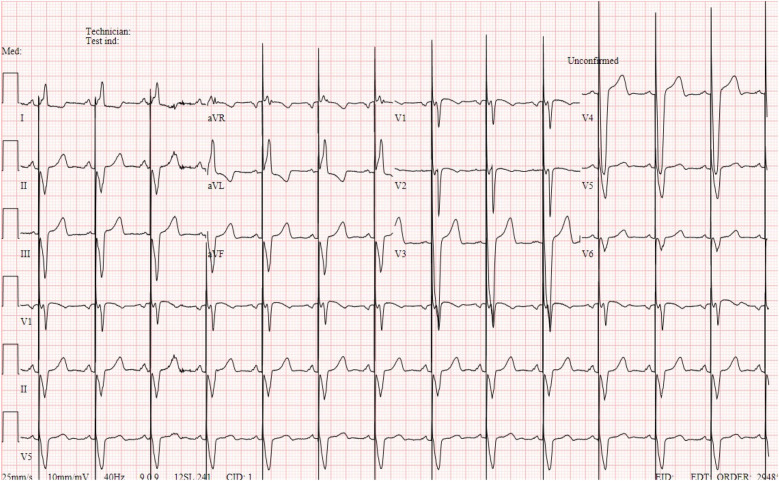
Electrocardiogram showing atrial sensing and paced ventricular rhythm with a QRS of 150 ms.

At the 1-month follow-up, echocardiography showed normal left ventricular size and function, no residual ventricular septal defect (VSD), and a right ventricular to pulmonary artery conduit (RV-PA) with a velocity of 2 m/s. Electrocardiogram showed paced ventricular rhythm and atrial sensing with a QRS of 150 ms (Figure 1).

Three months later, pacemaker interrogation showed an increase in ventricular unipolar lead impedance reaching 190 Ω, with an increase in capture threshold reaching 5.5 V requiring an increase in pacemaker output.

This right ventricular lead deterioration was observed during follow-up over the upcoming months with further increase in ventricular lead impedance and capture threshold requiring an increase in ventricular output.

A few months later, he underwent device change; however, during follow-up, there was persistence of right ventricular lead deterioration with an increase in capture threshold, which drained the device with an estimated longevity of <1 year.

Given the persistence of the right ventricular lead deterioration, a dual chamber transvenous pacemaker implantation with left bundle branch pacing was scheduled.

**Figure 2:**
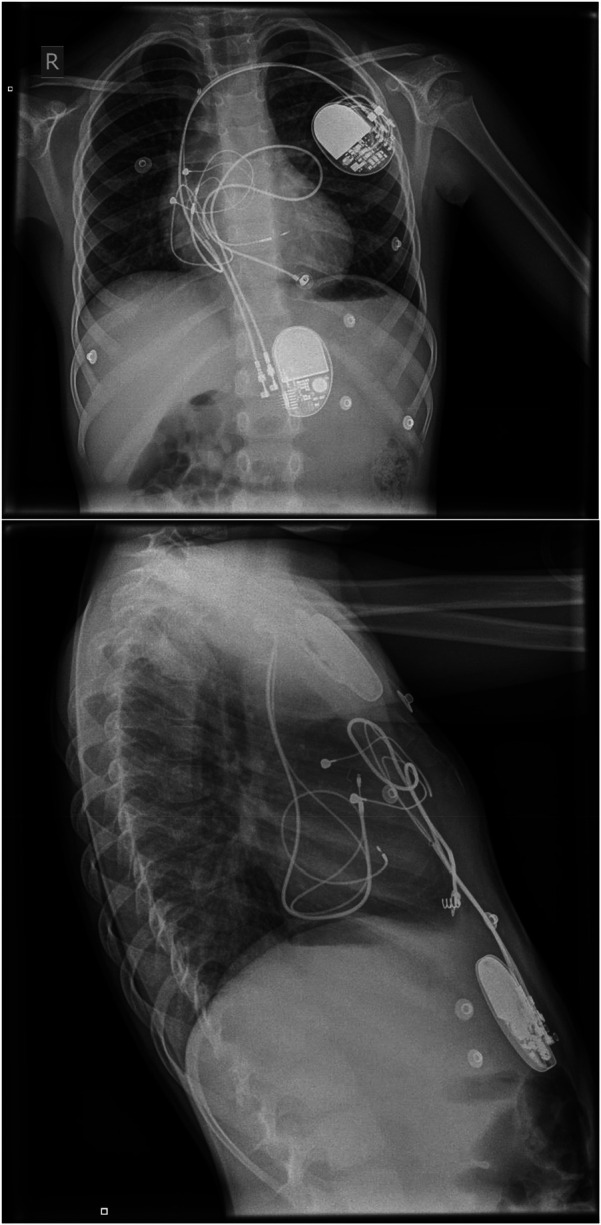
Posteroanterior (PA) and lateral x-ray chest showing a dual chamber pacemaker with its wire terminating in the right atrium and right ventricular septum. Another infradiaphragmatic dual chamber pacemaker with epicardial leads is also noted.

The patient underwent implantation of a dual chamber pacemaker (Sphera DR MRI, Medtronic). Using the delivery sheath (C31HIS, Medtronic), two leads were inserted via the left extra-thoracic subclavian vein. An atrial pacing lead was placed into the right atrium. The ventricular pacing lead (3830 Medtronic Selectsecure) was placed into the left bundle branch area at the middle part of the septum, where a W-pattern of the QRS complex was seen at V1 during pacing (Figure 2), achieving an LVAT of 75 ms and interpeak interval (V6-R’V1) of 50 ms. The lead was screwed with three slow rotations within the interventricular septum which was stopped once a right bundle branch block pattern was seen in lead V1 to avoid perforation.

**Figure 3:**
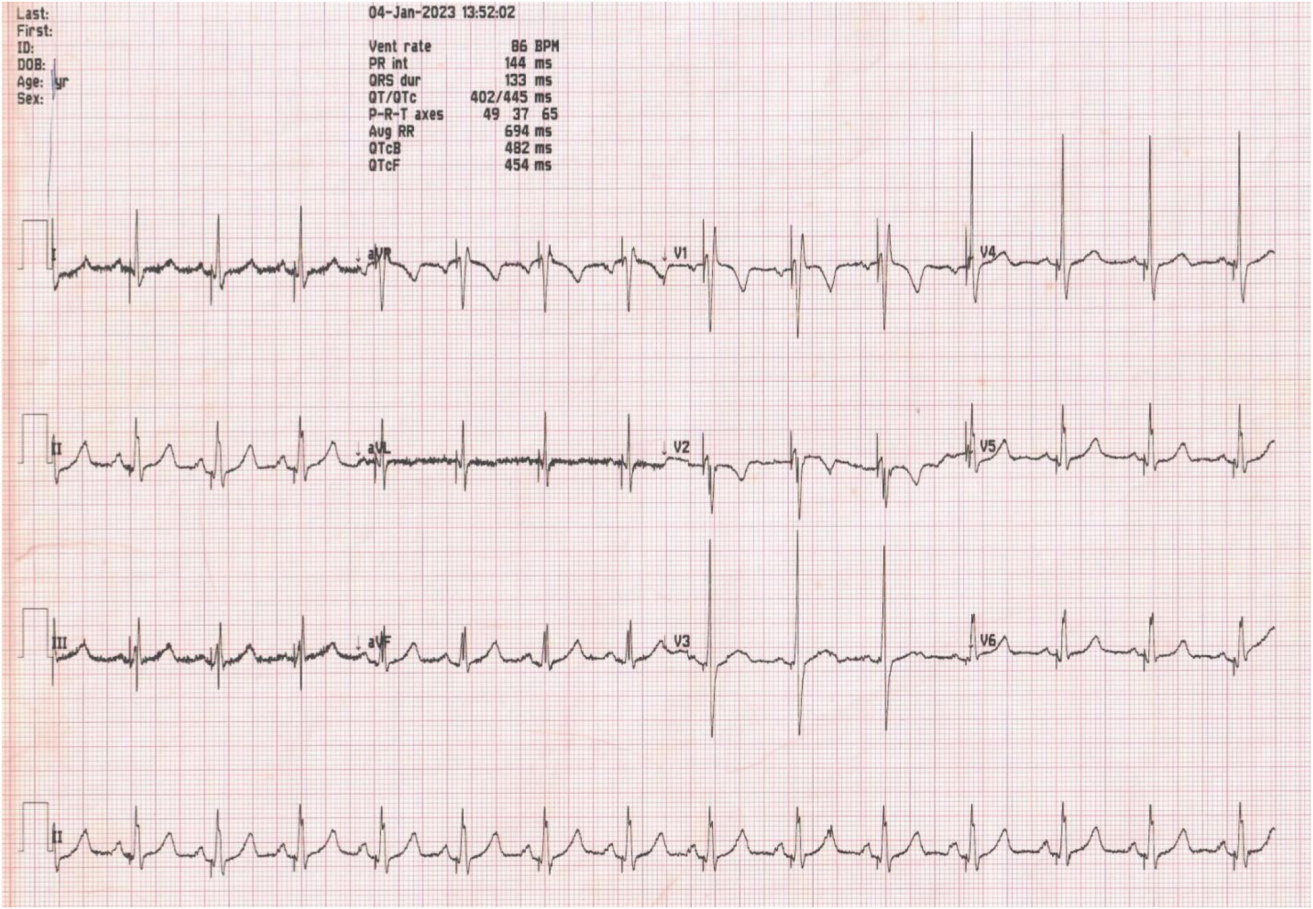
Electrocardiogram post dual chamber pacemaker with LBBP showing ventricular paced beats with a QRS of 100 ms.

Postoperative electrocardiogram showed a paced ventricular rhythm with a narrow QRS of 100 ms (Figure 3). The pacing threshold was 0.6 V at 0.4 ms, the R wave amplitude was 16 mV, and the pacing impedance was 550 Ω.

There were no complications noted during the perioperative period at 1-month, 3-month, 6-month, and 1-year follow-ups.

During follow-up visits, ECGs showed normal pacemaker function. Pacing parameters were stable, and echocardiography showed normal left ventricular ejection fraction (LVEF) (Supplementary Video S1).

## 3 Discussion

In recent years, the development of left bundle branch pacing (LBBP) has made it an incredibly desirable pacing technique. It has remedied several of the constraints of His bundle pacing (HBP), such as high and fluctuating capture thresholds, cross talk, and not fitting for distal lesions of the conduction system (4). All these advantages have turned LBBP into the most favored physiological pacing approach, particularly for ventricular pacing-dependent and congestive heart failure patients. Many cases of LBBP have been seen in adults; however, its use in kids is scarcely mentioned. Ponnusamy et al. (5) reported a 13-year-old girl who was born with a complete heart block and received selective LBBP, but its viability and security among smaller children are still not widespread. A retrospective analysis by Dai et al. (6) of six patients aged between 9 and 14 years, all of whom weighed between 26 and 48 kg, who received LBBP showed that it was both safe and doable for children in this age group.

This 4-year-old individual with corrected transposition of TGA that was complicated by complete heart block is one of the youngest who underwent dual chamber pacemaker implantation to date utilizing left bundle branch pacing (LBBP). It is significant to point out that the right ventricular pacing rate will likely be high, which may lead to cardiac insufficiency due to overstimulation of the right ventricle, particularly for a child. As such, physiological pacing should be the first choice. Thus, in this situation, we chose LBBP to correct the complete heart block and improve cardiac capacity due to the potential risk of an increased capture threshold of HBP (7). This approach helped to bypass the blocked area in the conduction by directly pacing the left bundle branch.

Usually, it might be difficult to perform a left bundle branch pacing (LBBP) procedure on a child, as the interventricular septum in children is generally thinner than in adults, but so far, the cases done on these children have proven to be safe and doable.

To note, one important difference between children and adults is that we need to make sure the lead is long enough for any potential growth; coiling it in the right atrium and tucking an extra length under the generator in the pocket might be an acceptable approach. Nevertheless, there is a potential for the coiled lead to generate fibrosis or adhere to the adjacent tissues. This is an issue worth monitoring, but there is no consensus on how to do so.

To summarize, this situation brings up the idea of employing LBBP as a pacing technique for younger kids with atrioventricular block. No issues appeared in the time leading up to and after the operation. The long-term results need to be monitored and assessed further.

This technique has proven so far to be a safe and practical one in young children, with the added benefits of a more physiological pacing and resolving some of the problems with other pacing methods.

## Data Availability

The original contributions presented in the study are included in the article/Supplementary Material, further inquiries can be directed to the corresponding author.
